# Protective Role of *Picralima nitida* Seed Extract in High-Fat High-Fructose-Fed Rats

**DOI:** 10.1155/2020/5206204

**Published:** 2020-10-24

**Authors:** Opeyemi Christianah De Campos, Daniel Ikpomwosa Osaigbovo, Titilayo Ifeoluwa Bisi-Adeniyi, Franklyn Nonso Iheagwam, Solomon Oladapo Rotimi, Shalom Nwodo Chinedu

**Affiliations:** ^1^Department of Biochemistry, College of Science and Technology, Covenant University, Canaan Land, PMB 1023, Ota, Ogun State, Nigeria; ^2^Covenant University Public Health and Wellbeing Research Cluster (CUPHERC), Covenant University, Canaan Land, PMB 1023, Ota, Ogun State, Nigeria

## Abstract

*Picralima nitida* is a therapeutic herb used in ethnomedicine for the management of several disease conditions including diabetes. This study examined the potential palliative effect of aqueous seed extract of *Picralima nitida* (APN) on dyslipidemia, hyperglycemia, oxidative stress, insulin resistance, and the expression of some metabolic genes in high-fat high-fructose-fed rats. Experimental rats (2 months old) were fed a control diet or a high-fat diet with 25% fructose (HFHF diet) in their drinking water for nine weeks. APN was administered orally during the last four weeks. Anthropometric and antioxidant parameters, lipid profile, plasma glucose, and insulin levels and the relative expression of some metabolic genes were assessed. APN caused a significant decrease (*P* < 0.05) in weight gained, body mass index, insulin resistance, plasma glucose, and insulin levels. High-density lipoprotein cholesterol level was significantly increased (*P* < 0.05), while triacylglycerol, cholesterol, low-density lipoprotein, cardiac index, atherogenic index, coronary artery index, and malondialdehyde levels in plasma and liver samples were also significantly decreased (*P* < 0.05) by APN at all experimental doses when compared to the group fed with an HFHF diet only. APN also significantly (*P* < 0.05) upregulated the relative expression of glucokinase, carnitine palmitoyltransferase-1 (CPT-1), and leptin at 400 mg/kg body weight when compared to the group fed with an HFHF diet only. This study showed that APN alleviated dyslipidemia, hyperglycemia, and oxidant effect associated with the intake of a high-fat high-fructose diet.

## 1. Introduction

Worldwide prevalence of various metabolic and cardiovascular disorders is still a major concern among health practitioners and researchers today [1, 2]. Metabolic syndrome categorized by a group of interrelated metabolic, clinical, and biochemical features has been shown to increase the susceptibility of an individual to cardiovascular disease and type 2 diabetes (T2DM). These features include increased weight gained, waist circumference, hyperglycemia, insulin resistance, and dyslipidemia [3–6]. In Africa, the incidence of metabolic syndrome is as high as 50%, depending on the population and the criteria used [7]. A recent study showed that the prevalence of metabolic syndrome among patients with type 2 diabetes was 59.62% in sub-Saharan Africa [8]. This is in contrast to what was obtainable in ancient times. A shift from the regular African/traditional diet to a western diet coupled with inadequate physical activity among several other factors might be responsible for the increasing rate of metabolic syndrome in Africa [1, 7, 9]. One major dietary lifestyle linked to metabolic syndrome and several cardiovascular disorders is the intake of high calorie-containing food [10]. This state of high-calorie malnutrition also results in oxidative stress [11, 12] which is a pathophysiological state characterized by a disparity in the number of antioxidants and oxidants produce in biological systems with the disparity favoring the oxidants [13].

Treatment and management of metabolic syndrome usually involve tackling all of the various risk factors classified under it. The focus is to prevent the progression of the risk factors to more complicated diseases [14, 15]. Several studies have shown the importance of plant-based food and botanicals in the treatment and management of metabolic syndrome caused by increased calorie intake [16–19].

*Picralima nitida* is a medicinal plant from the genus *Picralima* and plant family *Apocynaceae*. It is found in tropical African countries such as Ivory Coast, Nigeria, Uganda, and Gabon, and it is popularly known as *Abeere* in the Southwestern part of Nigeria among the Yoruba people [20–22]. The plant is used in traditional medicine for the treatment and management of malaria, abscesses, hepatitis, pneumonia, diabetes, and hypertension [21, 23]. The seeds are usually ground to a fine powder with the aid of a local grinder and added to foods [24] such as ogi (called pap in English) or taken as a decoction. Several studies have previously shown that various extracts of this plant are good sources of phytochemicals such as glycosides, alkaloids, triterpenes flavonoids, polyphenols, saponins, and tannins [23–29]. Alkaloids are the predominant bioactive compounds that have, so far, been isolated from the seeds of *P. nitida* [30]. Akuammicine, an indole alkaloid isolated from *P. nitida,* stimulated glucose uptake in differentiated 3T3-LI adipocytes. The report suggested that akuammicine could have acted singly or synergistically, with other bioactive compounds, to confer the accrued antidiabetic potential of the plant in folklore medicine [24]. Although few studies have corroborated the medicinal value of this plant, there is, however, a paucity of information on the scientific validation of the potential of this plant against high calorie-induced metabolic disorder and oxidative stress.

Hence, this present study examined the effect of aqueous seed extract of *Picralima nitida* (APN) on dyslipidemia, hyperglycemia, insulin resistance, and oxidative stress in high-fructose high-fat-fed rats.

## 2. Materials and Methods

### 2.1. Chemicals and Reagents

D-fructose was purchased from PhytoTechnology laboratories, Lenexa KS, The United States. A one-step RT-PCR kit (TransGen EasyScript®) was purchased from TransGen Biotech Co. Ltd (Beijing, China). All other chemicals, unless stated otherwise, including nicotinamide adenine dinucleotide phosphate (NADPH), sulphanilamide, 1-chloro-2,4-dinitrobenzene (CDNB), xylenol, 5.5-dithio bis 2-nitrobenzoic acid (DTNB), bovine serum albumin (BSA), pyrogallol, HEPES, N-(1-naphthyl) ethylenediamine, and reduced glutathione (GSH) were purchased from Sigma-Aldrich, Germany.

### 2.2. Preparation of Aqueous Seed Extract of *Picralima nitida*

*Picralima nitida* seeds were collected fresh from local farms in Ota, Ogun State, Nigeria. The plant was identified and validated at the Forestry Research Institute of Nigeria, Ibadan and specimen of the plant seed with voucher number FHI 111159 were deposited at the institute. The seeds were dehulled, air dried, crushed to powder using a blender, and extracted with distilled water for 72 h with a mass to volume ratio of 1 : 5 (g/L). The extract was dried under vacuum on a rotary evaporator at 55°C and stored at 4°C until use.

### 2.3. Laboratory Animals

Inbred male Wistar rats, Albinus, at two months old, were used in the study. The animals were made to adapt to the experimental and laboratory environmental conditions for two weeks in standard cages. The animals had free access to constant food and water and temperature in a reverse 12 h day/night cycle. The animals were maintained based on accepted guidelines following approval by the Covenant University Health Research Ethics Committee (CHREC/028/2018).

### 2.4. Experimental Design

All animals except the control animals (Group 1) were fed a high-fat diet (Table 1) with 25% fructose (HFHF) in their drinking water for 9 weeks. Blood glucose level was monitored via tail vein using Accu-Chek glucometer and Test Strip (Infopia Co., Ltd, South Korea), at 0, 2, 5, and 9 weeks. At the 5^th^ week, animals fed an HFHF diet and with a glucose level of ≥110 mg/dl were divided into four groups with one group (group 2) being fed an HFHF diet only throughout the experimental period. The other three groups, group 3, 4, and 5, were fed an HFHF diet throughout the experimental period with 100, 200, and 400 mg/kg body weight of *Picralima nitida* seed extract administered, respectively, via oral gavage during the last 4 weeks. The experimental doses were chosen based on a preliminary acute toxicity test which showed that the seed extract did not cause the death of experimental Wistar rats at 500, 1000, and 2000 mg/kg body weight. Also, from literature, the LD50 of various parts of the plant varied from 707.11 mg/kg to 14500 mg/kg with the highest LD50 value observed in experimental Wistar rats and the lowest in experimental mice [31–35]. Based on the fact that the LD50 is higher in Wistar rats, we decided to investigate the potential benefit of this plant at low to moderate doses.

### 2.5. Blood and Tissue Sampling

After 9 weeks, animals were weighed and anesthetized by intraperitoneal injection of 10 mg/kg of xylazine hydrochloride and 80 mg/kg of ketamine hydrochloride. Their length (nasal to anus length) and waist circumference was estimated using a standard measuring tape. Blood and tissue samples were prepared according to previously described methods [36].

### 2.6. Plasma Glucose and Insulin Level

Plasma glucose concentration was determined using the Randox glucose kit, Randox Laboratories Ltd, Crumlin, United Kingdom, while plasma insulin concentration was determined using Rat Insulin (INS) Enzyme-Linked Immunosorbent Assay (ELISA) kit, Hangzhou Eastbiopharma Co., Ltd, Hangzhou, China. The assays were carried out based on instructions outlined by the manufacturers. Insulin resistance was evaluated using the homeostasis model assessment of basal insulin resistance (HOMA-IR) and calculated as reported previously [11].

### 2.7. Lipid Profile

Plasma levels of triglycerides (TAG), total cholesterol (TC), and high-density lipoprotein cholesterol (HDLc) were analyzed using readily available commercial kits, Randox Laboratories Ltd., Crumlin, United Kingdom. Low-density lipoprotein cholesterol (LDLc) was calculated as reported previously [37]. Cardiac index (CI), atherogenic index (AI), and coronary artery index (CAI) were calculated as reported by [11] using the formulae below:

(1) $$\begin{array}{c} \text{CI }=\;\frac{\text{TC}}{\text{HDLc}}\text{,}\\ \text{AI }=\;\frac{\text{TC}-\text{HDLc}}{\text{HDLc}}\text{,}\\ \text{CAI }=\;\frac{\text{LDLc}}{\text{HDLc}}. \end{array}$$

### 2.8. Hepatic and Renal Function Assays

The activities of aspartate aminotransferase (ASP), alanine aminotransferase (ALT), alkaline phosphatase (ALP), and albumin levels in plasma samples were determined spectrophotometrically using Randox enzyme and albumin kits, Randox Laboratories Ltd, Crumlin, United Kingdom, based on instructions outlined in the manufacturer's guide. Urea and creatinine concentration in plasma and kidney homogenate samples were also assayed using Randox kit for urea and creatinine determination, Randox Laboratories Ltd., Crumlin, The United Kingdom.

### 2.9. Assessment of Oxidative Stress

The level of lipid peroxidation in plasma and liver homogenates was assessed by evaluating malondialdehyde (MDA) levels in samples using thiobarbituric acid reactive substances (TBARS) assay. Briefly, TBARS reagent (1.0 ml) containing 0.25 N HCL, 0.375% of thiobarbituric acid, and 15% of trichloroacetic acid was added to 50 *μ*l of plasma and liver homogenate samples. The mixture was heated at boiling point for 15 minutes in a water bath, cooled on ice, and centrifuged at 10,000 rpm for 10 min. The absorbance of the supernatant was recorded at 535 nm against a blank which contained all the reagents with distilled water replacing the samples. MDA levels were calculated from the extinction coefficient (1.56 × 10^6^) of the MDA-TBA complex [38].

Reduced glutathione level in plasma and liver homogenates was assayed according to a previously described method [39]. Glutathione-s-transferase (GST) activity in plasma and liver homogenate samples was evaluated based on glutathione-s-transferase catalyzed reaction of reduced glutathione (GSH) with 1-chloro-2,4-dinitrobenzene (CDNB) to give a thioether (S-2,4-dinitrophenyl glutathione) which can be monitored by an increased change in absorbance at 340 nm [40]. Superoxide dismutase (SOD) activity was assayed based on the ability of the enzyme to prevent auto-oxidation of pyrogallol [41].

### 2.10. Total Protein Determination

The total protein level in plasma and liver homogenate samples was determined by a previously described method [42].

### 2.11. Extraction of RNA and mRNA Expression of Some Metabolic Genes

RNA was extracted from liver samples using TRIpure (Aidlab, Biotechnologies Ltd, Beijing, China) isolation reagent based on the instruction outlined in the manufacturer's guide. The level of expression of some metabolic genes was assessed according to a previously described method [36]. Briefly, Easyscript one-step RT-PCR supermix kit (TransGen Biotech Co., Ltd., Beijing, China) was used for the semiquantitative process based on the manufacturer's instruction. cDNA was first synthesized by incubating the RNA template (500 ng) at 45^o^C for 30 minutes. A thermal cycler (C100 Touch thermal cycler, Bio-rad Laboratories) was used to carry out the amplification process using gene-specific primers as listed in Table 2. The PCR conditions included an initial denaturation at 94°C for 5 minutes, followed by 45 cycles of 94°C for 30 seconds, another 30 seconds at an annealing temperature of gene-specific primers and 1 min at 72°C. The PCR products were run on an ethidium bromide-stained agarose gel (1.5%) in Tris Borate EDTA buffer and viewed under UV light (UVP BioDoc-It™ Imaging system (Upland, CA, USA). The intensity of the bands was analyzed using Image J software [43]. Results are expressed as the mean ratio of the intensity of each gene to that of two reference genes (GAPDH and *β*-actin).

### 2.12. Statistical Analysis

Data generated were analyzed using statistical package for the social sciences (SPSS) (ver. 20.0, SPSS Inc., Chicago, IL, USA) and results were represented as mean ± SEM of at least five biological replicates. The level of heterogeneity among groups was assessed at *P* < 0.05 by one-way analysis of variance (ANOVA) followed by Duncan's multiple range test.

## 3. Results

### 3.1. Food Intake, Water Intake, and Effect of APN on Anthropometric Data, Plasma Glucose, and Insulin Level

Findings from this study showed that the group fed the control diet had increased mean daily food and water intake when compared with those fed an HFHF diet only. Despite this, rats fed with the HFHF diet had increased weight gained, body mass index (BMI), and lee index after 9 weeks. APN, however, significantly reduced (*P* < 0.05) weight gained and BMI at all the experimental dose used in this study when compared to those fed with HFHF diet only (Table 3). This reduction was, however, not significant when compared to the control group.

Fasting plasma glucose and insulin levels of the group fed an HFHF diet only was significantly increased when compared to those fed the control diet (Table 3). APN significantly reduced (*P* < 0.05) the effect of HFHF diet on plasma glucose concentration at all the doses used in this study while plasma insulin level, on the other hand, was significantly reduced (*P* < 0.05) at 100 mg/kg body weight of APN. There was, however, no significant difference in plasma glucose and insulin concentrations among the groups fed an HFHF diet and treated with APN when compared to the control group (Table 3).

Insulin resistance calculated based on homeostasis model assessment was found to increase significantly (*P* < 0.05) in HFHF-fed rats in comparison with those fed the control diet. APN significantly reduced insulin resistance at the various doses of the extracts. There was, however, no significant difference in insulin resistance among the treated groups administered APN when compared to the groups fed with only the control diet (Table 3).

### 3.2. Effect of APN on Lipid Profile

**Figure 1:**
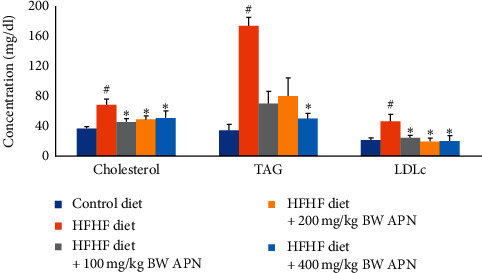
Effect of APN on cholesterol, triacylglycerol, and low-density lipoprotein cholesterol of rats fed an HFHF diet. Values are represented as mean ± SEM of at least five biological replicates. Bars with # are significantly (*P* < 0.05) different from control while bars with ^*∗*^ are significantly different (*P* < 0.05) from HFHF-fed group.

Plasma HDL levels of the group fed an HFHF diet, in comparison to the control group, were significantly decreased (*P* < 0.05). APN, at all experimental doses, ameliorated the effect of the HFHF diet by causing a significant increase (*P* < 0.05) in HDL levels. The ameliorative effect was dose-dependent (Figure 1).

**Figure 2:**
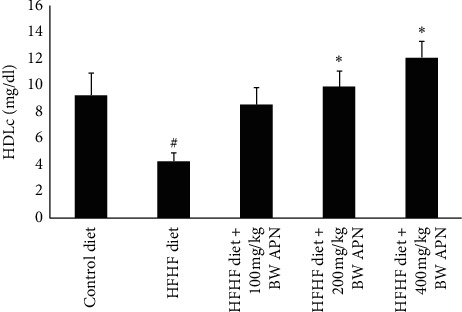
Effect of APN on HDLc of rats fed an HFHF diet. Values are represented as mean ± SEM of at least five biological replicates. Bars with # are significantly (*P* < 0.05) different from control while bars with ^*∗*^ are significantly different (*P* < 0.05) from HFHF-fed group.

Triacylglycerol, total cholesterol, and LDL cholesterol levels of rats fed an HFHF diet were significantly increased (*P* < 0.05) when compared to those fed a control diet (Figure 1). Coronary artery index, cardiac index, atherogenic index, and coronary artery index of the group fed the HFHF diet were also significantly increased (*P* < 0.05) when compared to those fed with the control diet (Figure 2). APN significantly reduced (*P* < 0.05) total cholesterol and LDL cholesterol levels of HFHF-fed rats at all experimental doses of the extract used in this study (Figure 1). Triglyceride levels were also reduced. The reduction was, however, only significant (*P* < 0.05) at the highest administered dose of APN. APN also caused a significant reduction in cardiac index, atherogenic index, and coronary artery index at all experimental doses of APN when compared with the group fed the HFHF diet only (Figure 2).

### 3.3. Effect of APN on Hepatic and Renal Functions

Findings from this study also showed that in comparison with the group fed the control diet, ALP and ALT activities were significantly increased (*P* < 0.05) in the group fed an HFHF diet only. APN, however, significantly reduced ALP and ALT activities. There were no significant differences in plasma albumin, urea, plasma creatinine, and kidney creatinine levels of rats-fed with HFHF diet and treated with APN when compared with the control and HFHF-fed groups (Table 4).

### 3.4. Effect of APN on Lipid Peroxidation, GSH Level, GST, and SOD Activity

**Figure 3:**
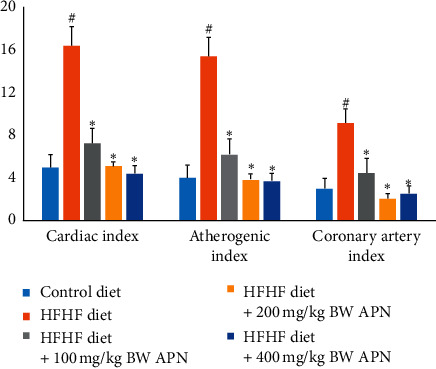
Effect of APN on the cardiac index, atherogenic index, and coronary artery index of rats fed an HFHF diet. Values are represented as mean ± SEM of at least five biological replicates. Bars with # are significantly (*P* < 0.05) different from control while bars with ^*∗*^ are significantly different (*P* < 0.05) from HFHF-fed group.

**Figure 4:**
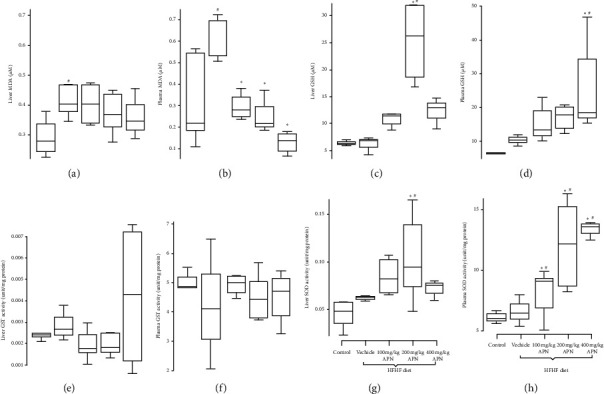
Effect of APN on MDA concentration and antioxidant parameters of rats fed an HFHF diet. (a) MDA level in liver homogenates; (b) MDA level in plasma samples; (c) GSH level in liver homogenates; (d) GSH level in plasma samples; (e) GST activity in liver homogenates; (f) GST activity in plasma; (g) SOD activity in liver homogenates; (h) SOD activity in plasma samples Bars with ^#^ are significantly (*P* < 0.05) different from control while bars with ^*∗*^ are significantly different (*P* < 0.05) from HFHF-fed group.

MDA levels increased significantly (*P* < 0.05) in plasma and liver homogenates of rats fed with an HFHF diet only when compared to those fed with the control diet (Figure 3). APN was, however, able to significantly (*P* < 0.05) reduce the oxidant effect of HFHF diet. APN also significantly increased (*P* < 0.05) the concentration of GSH in plasma and liver homogenates at 400 mg/kg BW and 200 mg /kg BW, respectively, when compared to the group fed the control diet or HFHF diet (Figure 3). SOD activity in liver samples of the groups fed an HFHF diet and treated with different doses of APN was significantly increased when compared to those fed a control diet or HFHF diet only (Figure 4).

### 3.5. Effect of APN on the Relative Expression of Some Metabolic Genes

**Figure 5:**
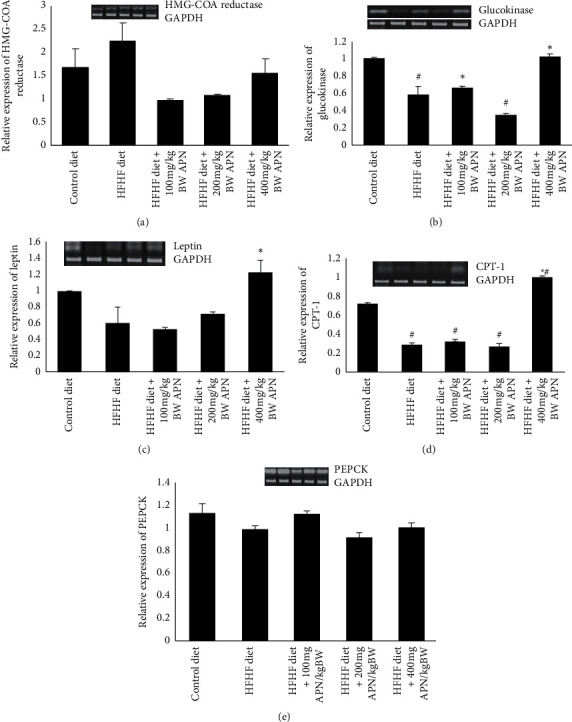
Effect of APN on the relative expression of some metabolic genes. (a) Relative expression of HMG-COA reductase; (b) relative expression of glucokinase; (c) relative expression of leptin; (d) relative expression of CPT-1; (e) relative expression of PEPCK. *β*-Actin and GAPDH were used as the reference gene to calculate the relative expression of the genes. Bars with ^#^ are significantly (*P* < 0.05) different from control while bars with ^*∗*^ are significantly different (*P* < 0.05) from HFHF-fed group.

The relative expression of genes coding for HMG-COA reductase, glucokinase, phosphoenolpyruvate carboxykinase (PEPCK), leptin, and carnitine palmitoyltransferase 1 were assessed in the liver of control and all treatment groups (Figure 4). The result showed that consumption of the HFHF diet led to a 35% and a 16% increase in the relative expressions of HMG-COA reductase and PEPCK when compared to those fed the control diet. APN, however, downregulated the expression of HMG-COA reductase at all experimental doses used in this study (Figure 4). The relative expression of glucokinase and carnitine palmitoyltransferase-1 (CPT-1) was significantly (*P* < 0.05) downregulated in the group fed an HFHF diet when compared to those fed the control diet. APN, however, significantly upregulated the expression of glucokinase at 100 and 400 mg/kg body weight while the relative expression of CPT-1 was only significantly upregulated at 400 mg/kg body weight of APN (Figure 4). The relative expression of leptin was downregulated by 39% in rats fed an HFHF diet when compared to those fed the control diet. APN, however, upregulated the expression when compared to the control group (Figure 5).

## 4. Discussion

The increasing prevalence of metabolic-related disorders and the morbidity and mortality associated with them has spurred a growing interest in identifying and validating botanicals that can help stem the tide. This research work investigated the potential palliative role of aqueous seed extract of *Picralima nitida* on weight gained, lee index, BMI, hyperglycemia, dyslipidemia, and oxidative stress in rats fed a high fructose high-fat diet. Weight gained, lee index, and body mass index (BMI) are common anthropometric measures of obesity used in most experimental rodents studies [44]. Although rats fed an HFHF diet only had decreased mean daily food and water intakes, they gained more weight and had higher BMI, plasma fasting insulin and glucose levels than those fed the control diet. Elevated plasma glucose and insulin observed among the group fed only an HFHF diet suggest a state of insulin insensitivity which could result from the inability of muscle and liver cells to take up glucose. Dyslipidemia characterized by an increase in TAG, TC and LDLc followed by a decrease in HDLc levels was also observed among the group fed an HFHF diet only. These metabolic modifications observed in this study substantiated the claim in experimental models that high calorie-containing foods cause glucose levels to rise in the blood, insulin insensitivity and dyslipidemia all of which increases the development of cardiovascular related disorders [11, 16, 45–49]. The ability of APN to reduce plasma glucose levels is in line with previous studies that showed its hypoglycemic effect *in vitro* and *in vivo* [23, 31, 35, 50]. Also, the capability of the plant extract to reduce the weight gained and body mass index shows its antiobesity potential while its ability to reduce cholesterol, triacylglycerol, and LDLc while increasing HDLc show its antidyslipidemic action and suggests that the plant could offer protective action against cardiovascular diseases.

The expression of some carbohydrate and lipid metabolizing enzymes in the liver of all treatment groups was also examined. Findings from this study showed that consumption of HFHF diet caused a downregulation of the expression of hepatic glucokinase and a mild upregulation of phosphoenolpyruvate carboxykinase when compared to that of the control. In the liver, glucokinase plays a critical catalytic role by promoting the addition of a phosphate group to glucose, thereby enhancing its uptake in the liver [51, 52]. Phosphoenolpyruvate carboxykinase, on the other hand, is a crucial enzyme in the gluconeogenesis pathway that helps to increase glucose production [53]. Reduced expression of glucokinase in the liver may be a result of the elevated plasma glucose concentration observed in rats fed an HFHF diet only indicating an inability to promote glucose uptake in the liver despite elevated insulin levels. APN, however, helped to upregulate the expression of glucokinase at 100 and 400 mg/kg body weight, demonstrating that APN can increase the expression of glucokinase and thus promote glucose uptake by the liver. Activators of glucokinase have been shown to be important in the treatment of diabetes [52, 54]. The mRNA expression level of carnitine palmitoyltransferase 1 *α* (CPT-1*α*), an important enzyme that aids the transport of long-chain free fatty acids into the mitochondria where they undergo *β*-oxidation, has been shown to decrease in experimental rodents fed a high-fat diet, high fructose diet, or high-fat high fructose combined diet [53, 55–57]. Conversely, mRNA expression of HMG-COA reductase, a rate-limiting enzyme of the cholesterol biosynthetic pathway, has been reported to increase following the consumption of a high-fat diet in rats [58, 59]. These reports corroborate findings from this study which indicated that intake of a high fructose high-fat diet caused a significant downregulation of the expression of CPT-1*α* while causing an upregulation in the expression levels of HMG-COA reductases. The aqueous seed extract of *Picralima nitida,* however, modulates the expression levels of these lipid metabolizing genes by downregulating the expression of HMG-COA- reductase at all experimental doses and upregulating the expression of CPT-1*α* at the highest dose of the extract. Surprisingly relative expression of leptin was downregulated in rats fed an HFHF diet when compared to the control group. APN, however, upregulated the relative expression of leptin at 400 mg/kg body weight. Although leptin is known to promote satiety, in the liver, it is known to prevent the synthesis of fat while promoting the breakdown of fatty acids [60].

Intake of high-fat high fructose diet has also been linked to hepatic dysfunction characterized by elevated levels of plasma ALT, AST, and ALP [19, 45, 61, 62]. Similarly, results from this study showed consumption of an HFHF diet caused a significant increase in plasma ALT and ALP levels when compared to the control diet. These enzymes are critical hepatic enzymes that leak into the bloodstream when the liver is damaged or injured and have served as critical biomarkers for assessing hepatic dysfunction. APN, however, significantly decreases the plasma level of these enzymes emphasizing its hepatoprotective potential.

Several reports have also shown a strong correlation between features of metabolic syndrome and oxidative stress [13, 61, 63, 64]. It is well-known that a high intake of fat- and carbohydrate-containing food coupled with an inactive lifestyle creates an imbalance in the energy status of the body [65, 66]. This often leads to an elevated amount of glucose in the blood, which are often stored as fats in the adipose tissues [64]. An elevated level of lipids/fats causes a cascade of reactions that promotes the formation of lipid peroxides via the process of lipid peroxidation, which are highly reactive and ultimately damage cells and tissues [67]. The level of lipid peroxidation in most biological samples is usually assessed by determining the malondialdehyde level (MDA) in such samples. This study, like previous studies [19, 48], showed that rats fed an HFHF had elevated levels of malondialdehyde in liver homogenates and plasma samples when compared to those fed the control diet. The ability of APN to cause a reduction in MDA level can be attributed to its antihyperlipidemic action as observed in this study.

Interestingly, APN also significantly increased GSH level and SOD activity in plasma and liver samples, suggesting a protective action of the plant against reactive oxygen species. GSH is a nonenzymatic antioxidant and a major low molecular weight thiol in most animal cells [68]. It plays a critical role, directly or indirectly, in scavenging reactive oxygen and nitrogen species. SOD, on the other hand, is an essential antioxidant enzyme that helps in neutralizing the harmful effect of superoxide ion by converting it to oxygen and hydrogen peroxide [69].

Although this study did not focus on the bioactive compounds present in this plant, several studies have previously shown that various extracts of this plant are good sources of phytochemicals [26, 27, 30, 33, 70]. More specifically, the seed extract of the *P. nitida* has been reported to contain glycosides, alkaloids, triterpenes flavonoids, polyphenols, saponins, and tannins [31, 33, 35]. Akuammicine, indole alkaloid isolated from the seeds of *P. nitida,* was reported to be effective in stimulating glucose uptake in differentiated 3T3-LI adipocytes [24]. The protective role of the APN observed in this study can thus be attributed to the phytochemicals present in the plant. These compounds may act singly or synergistically to confer their effects.

## 5. Conclusions

In conclusion, this study showed that APN alleviated dyslipidemia, hyperglycemia, and pro-oxidant status associated with the intake of a high-fat high fructose diet.

## Figures and Tables

**Table 1:** Feed and diet composition.

	Control diet (g/kg)	HCHF (g/kg)
Corn starch	450	200
Beef tallow	–	250
Sucrose	100	100
Cellulose	100	100
Soya bean oil	70	70
Fish meal	240	240
Vitamin and mineral mix^a^	40	40

^a^Contained (per kg diet) vitamin A (4 000 000 IU), Vitamin D3 (8 00 000 IU), vitamin E (8 000 IU), vitamin K3 (0.9 g), vitamin B1 (0.7 g), vitamin B2 (2 g), vitamin B6 (1.2 g) vitamin B12 (0.006 g), nicotinic acid (11 g), panthethoic acid (3 g) folic acid (3 g) biotin (0.02 g) choline 120 g, CuSO_4_·5H_2_O (2 g) CoCl_2_·6H_2_O (0.008), NaCl (2 g), FeSO_4_·7H_2_O (8 g), KI (0.48 g), MnSO_4_·7H_2_O (32 g), CaSO_4_ (14 g), and ZnSO_4_ (20 g).

**Table 2:** Sequences of gene-specific primers.

Gene	Sequence (5′-3′)	Template
GAPDH	Forward AGTGCCAGCCTCGTCTCATA	NM_017008.4
	Reverse GATGGTGATGGGTTTCCCGT	
*β*-Actin	Forward: GTCAGGTCATCACTATCGGCAAT	NM_031144.3
	Reverse: AGAGGTCTTTACGGATGTCAACGT	
GK	Forward: CATATGTGCTCCGCAGGACTA	NM_001270850.1
	Reverse: CTTGTACACGGAGCCATCCA	
PEPCK	Forward: AGCCTCGACAGCCTGCCCCAGG	NM_198780.3
	Reverse: CCAGTTGTTGACCAAAGGCTTT	
HMG-COA reductase	Forward: TGCTGCTTTGGCTGTATGTC	NM_013134.2
	Reverse: TGAGCGTGAACAAGAACCAG	
CPT-1*α*	Forward: AAGTCAACGGCAGAGCAGAG	NM_031559.2
	Reverse: ACGCCCAAGTATTCACAGGG	
Leptin	Forward: GCCAAGGCAAACCCATTCTG	XM_008762762.2
	Reverse: GATACCGACTGCGTGTGTGA	

GAPDH = Glyceraldehyde 3-phosphate dehydrogenase; GK = glucokinase; PEPCK= Phosphoenolpyruvate carboxykinase; HMG-COA reductase = 3-hydroxy-3-methyl-glutaryl-coenzyme A reductase; CPT-1*α* = carnitine palmitoyltransferase 1*α*

**Table 3:** Effect of APN on weight gained, lee index, BMI, fasting plasma glucose and insulin level, and insulin resistance.

	Control diet	HFHF diet	HFHF + 100 mg/kg BW APN	HFHF + 200 mg/kgBW APN	HFHF + 400 mg/kgBW APN
Food intake (g)	18.50 ± 0.57	14.93 ± 0.69^#^	12.30 ± 0.69^#^	12.33 ± 1.04^#^	13.25 ± 1.11^#^
Water/high fructose (ml)	31.98 ± 0.66	22.97 ± 1.10^#^	20.62 ± 1.21^#^	21.03 ± 1.11^#^	22.33 ± 1.31^#^
Weight gained at 9 weeks (g)	57.50 ± 7.52	70.33 ± 5.36	39.17 ± 2.50^*∗*^	42.80 ± 3.95^*∗*^	51.00 ± 9.01^*∗*^
BMI (g/cm^2^)	0.55 ± 0.02	0.65 ± 0.02^#^	0.57 ± 0.02^*∗*^	0.52 ± 0.01^*∗*^	0.54 ± 0.02^*∗*^
Lee index	0.294 ± 0.006	0.313 ± 0.005	0.314 ± 0.013	0.294 ± 0.05	0.299 ± 0.007
Plasma glucose (mmol/l)	7.57 ± 1.05	11.15 ± 0.58^#^	6.80 ± 0.63^*∗*^	7.16 ± 0.48^*∗*^	6.37 ± 1.07^*∗*^
Insulin level (mlU/L)	7.78 ± 1.21	10.53 ± 0.61^#^	7.37 ± 1.16^*∗*^	9.62 ± 0.21	9.59 ± 0.51
HOMA-IR	2.77 ± 0.64	5.40 ± 0.56^#^	2.13 ± 0.29^*∗*^	3.09 ± 0.20^*∗*^	2.66 ± 0.38^*∗*^

Values are represented as mean ± SEM. Values on the same row with # are significantly (*P* < 0.05) different from control while those with ^*∗*^ are significantly different (*P* < 0.05) from the HFHF-fed group.

**Table 4:** Effect of APN on the liver and kidney function.

	Control diet	HFHF diet	HFHF + 100 mg/kg BW APN	HFHF + 200 mg/kgBW APN	HFHF + 400 mg/kgBW APN
Liver weight (g)	7.25 ± 0.28	8.23 ± 0.53	7.08 ± 0.71	6.27 ± 0.14^*∗*^	6.75 ± 0.43^*∗*^
Kidney weight (g)	2.57 ± 0.31	2.76 ± 0.17	2.90 ± 0.31	2.90 ± 0.23	2.40 ± 0.18
Total protein (mg/ml)	55.67 ± 0.76	73.31 ± 2.52	68.80 ± 3.63	64.64 ± 5.76	83.65 ± 6.23^#^
ALP (U/l)	117.76 ± 10.78	150.88 ± 22.17	111.09 ± 8.80	74.70 ± 8.69^*∗*^	84.64 ± 6.46^*∗*^
AST (U/l)	25.04 ± 0.48	34.32 ± 1.33	24.46 ± 3.11	30.76 ± 4.60	29.39 ± 5.38
ALT (U/l)	41.43 ± 2.95	62.94 ± 4.26^#^	26.80 ± 5.57	17.81 ± 1.75^*∗#*^	18.82 ± 0.85^*∗#*^
Albumin (U/l)	4.63 ± 0.16	4.01 ± 0.16	5.09 ± 0.54	4.64 ± 0.47	3.82 ± 0.58
Plasma urea (mg/dl)	61.87 ± 1.75	62.69 ± 4.24	70.69 ± 5.14	65.33 ± 2.64	69.39 ± 3.51
Kidney urea (mg/dl	32.87 ± 4.21	42.29 ± 4.85	50.04 ± 6.54	52.70 ± 6.37^#^	49.69 ± 2.64
Plasma creatinine (mg/dl)	3.64 ± 0.19	3.63 ± 0.51	3.33 ± 0.23	3.41 ± 0.21	3.17 ± 0.07
Kidney creatinine (mg/dl)	2.45 ± 0.21	2.31 ± 0.30	2.18 ± 0.13	1.97 ± 0.14	2.80 ± 0.85

Values are represented as mean ± SEM. Values on the same row with ^#^ are significantly (*P* < 0.05) different from control while those with ^*∗*^ are significantly different (*P* < 0.05) from HFHF-fed group.

## Data Availability

All data have been included in the article.
